# Handoffs and transitions in critical care (HATRICC): protocol for a mixed methods study of operating room to intensive care unit handoffs

**DOI:** 10.1186/1471-2482-14-96

**Published:** 2014-11-19

**Authors:** Meghan B Lane-Fall, Rinad S Beidas, Jose L Pascual, Meredith L Collard, Hannah G Peifer, Tyler J Chavez, Mark E Barry, Jacob T Gutsche, Scott D Halpern, Lee A Fleisher, Frances K Barg

**Affiliations:** Department of Anesthesiology and Critical Care, Perelman School of Medicine, University of Pennsylvania, 3400 Spruce Street, 680 Dulles Building, Philadelphia, PA 19104 USA; Center for Healthcare Improvement and Patient Safety, Department of Medicine, Perelman School of Medicine, University of Pennsylvania, 1209 Blockley Hall, 423 Guardian Drive, Philadelphia, PA 19104 USA; Leonard Davis Institute of Health Economics, University of Pennsylvania, Colonial, Penn Center, 3641 Locust Walk, Philadelphia, PA 19104 USA; Department of Psychiatry, Perelman School of Medicine, University of Pennsylvania, 3535 Market Street, Philadelphia, PA 19104 USA; Department of Surgery, Division of Traumatology, Surgical Critical Care and Emergency Surgery, Perelman School of Medicine, University of Pennsylvania, 3400 Spruce Street, 5 Maloney Building, Philadelphia, PA 19104 USA; School of Nursing, University of Pennsylvania, Philadelphia, PA 19104 USA; School of Arts and Sciences, University of Pennsylvania, Philadelphia, PA USA; College of Arts and Sciences, New Mexico State University, Las Cruces, NM 88003 USA; Department of Medicine, Division of Pulmonary, Allergy and Critical Care Medicine, Perelman School of Medicine, University of Pennsylvania, 719 Blockley Hall, 423 Guardian Drive, Philadelphia, PA 19104 USA; Center for Clinical Epidemiology and Biostatistics, Perelman School of Medicine, University of Pennsylvania, Philadelphia, PA 19104 USA; Department of Family Medicine, Perelman School of Medicine, University of Pennsylvania, 3620 Hamilton Walk, Philadelphia, PA 19104 USA; Department of Medical Anthropology, School of Arts and Sciences, University of Pennsylvania, Philadelphia, PA 19104 USA

**Keywords:** Implementation, Quality improvement, Patient safety, Handoffs, Intensive care unit, Postoperative care, Simulation

## Abstract

**Background:**

Operating room to intensive care unit handoffs are high-risk events for critically ill patients. Studies in selected patient populations show that standardizing operating room to intensive care unit handoffs improves information exchange and decreases errors. To adapt these findings to mixed surgical populations, we propose to study the implementation of a standardized operating room to intensive care unit handoff process in two intensive care units currently without an existing standard process.

**Methods/Design:**

The Handoffs and Transitions in Critical Care (HATRICC) study is a hybrid effectiveness- implementation trial of operating room to intensive care unit handoffs. We will use mixed methods to conduct a needs assessment of the current handoff process, adapt published handoff processes, and implement a new standardized handoff process in two academic intensive care units. *Needs assessment*: We will use non-participant observation to observe the current handoff process. Focus groups, interviews, and surveys of clinicians will elicit participants’ impressions about the current process. *Adaptation and implementation*: We will adapt published standardized handoff processes using the needs assessment findings. We will use small group simulation to test the new process’ feasibility. After simulation, we will incorporate the new handoff process into the clinical work of all providers in the study units. *Evaluation*: Using the same methods employed in the needs assessment phase, we will evaluate use of the new handoff process. *Data analysis*: The primary effectiveness outcome is the number of information omissions per handoff episode as compared to the pre-intervention period. Additional intervention outcomes include patient intensive care unit length of stay and intensive care unit mortality. The primary implementation outcome is acceptability of the new process. Additional implementation outcomes include feasibility, fidelity and sustainability.

**Discussion:**

The HATRICC study will examine the effectiveness and implementation of a standardized operating room to intensive care unit handoff process. Findings from this study have the potential to improve healthcare communication and outcomes for critically ill patients.

**Trial registration:**

ClinicalTrials.gov identifier: NCT02267174. Date of registration October 16, 2014.

**Electronic supplementary material:**

The online version of this article (doi:10.1186/1471-2482-14-96) contains supplementary material, which is available to authorized users.

## Background

Handoffs are transfers of patient care and accountability that are a well-recognized risk factor for adverse events in healthcare such as medication errors [1] and delays in diagnosis or treatment [2]. Handoffs are ubiquitous in medicine due to the specialization of care across providers, disciplines and care settings [3]. Despite mounting evidence that handoff standardization is beneficial [4–13], variability in handoff processes persists in all the areas associated with surgical and anesthetic practice [14]. Of particular concern is the handoff occurring for patients admitted from the operating room (OR) to the intensive care unit (ICU). This transfer is high risk because it involves physical movement of patients and multiple handoffs among providers of different disciplines (anesthetist to critical care clinician, surgeon to critical care clinician, operating room nurse to critical care nurse, *et cetera*) [15]. Also, the patients whose care is transferred are often incapacitated and thus unable to participate, making them vulnerable to error and preventable harm.

Published reports demonstrate improved information exchange [5, 7–12] and improved patient outcomes [4, 9] when *cardiac* OR-to-ICU handoffs are standardized, especially in pediatric cardiac populations [7–12]. Though the findings from this work are intriguing, it is unclear whether the approaches adopted in postoperative pediatric cardiac care apply to *all* patients requiring postoperative critical care. Heterogeneous patient groups have differing levels of acuity and different nursing needs. Additionally, a larger number of potential procedures and providers may complicate the adoption of and adherence to a standard process. The proposed project aims to adapt previously published approaches to handoff standardization and to implement a handoff process that will be applicable to the care of patients undergoing cardiac, general surgical, orthopedic, transplant, trauma, and vascular surgical procedures.

The Handoffs and Transitions in Critical Care (HATRICC) study will employ a mixed methods hybrid effectiveness-implementation design [16] to study both the implementation and effectiveness of a standard OR-to-ICU handoff process in a mixed surgical population. As Curran *et al.* discussed in their work describing hybrid study models, this study design has the potential to speed the adoption of evidence based practice by providing information on the effectiveness of interventions in real world settings while simultaneously collecting information about implementation strategies [16]. There is sufficient evidence to support a trial of handoff standardization in our study population, but we do not know whether standardization will be effective for a mixed surgical population. The procedures needed to effectively implement a standard postoperative handoff process are similarly unclear.

### Project aims and hypotheses

There are multiple surgical settings with demonstrated inadequate handoff practices: intraoperative handoffs between anesthesia providers, [17, 18] handoffs in the post-anesthesia care unit, [19] handoffs from the ICU to the OR, [20] and those from the OR to the ICU [5, 7–12]. We chose to study OR-to-ICU handoffs because the evidence base supporting handoff standardization is more established for these handoffs than for the others mentioned. The HATRICC study has three aims:

1. Perform a needs assessment of the OR-to-ICU handoff process in two ICUs that serve mixed surgical populations.
2. Adapt and implement a standardized OR-to-ICU handoff process.
3. Evaluate the implementation and effectiveness of a standardized OR-to-ICU handoff process.

The project will test both intervention effectiveness and implementation hypotheses:

*Intervention effectiveness hypothesis:* After implementing a standard handoff process, the number of information omissions per handoff will decrease by 50%.

*Implementation hypothesis:* Clinician acceptance of a new standardized OR-to-ICU handoff process will be high, as assessed qualitatively.

**Figure 1:**
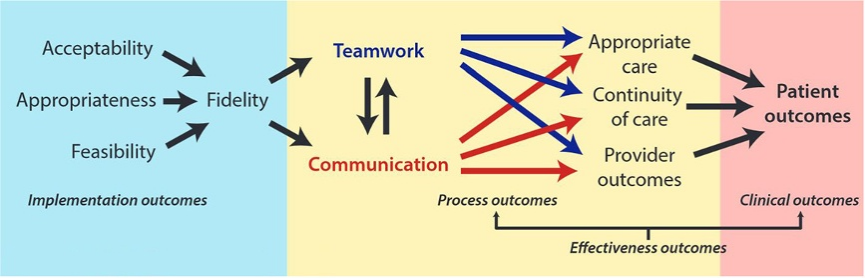
**HATRICC conceptual model.**

HATRICC is based on a conceptual model that relates teamwork and communication to patient outcomes (Figure 1). In addition to addressing the three aims above, we will use study measures to test whether the relationships in this model are consistent with our observations of actual clinical practice.

## Methods/Design

HATRICC is a Type 1 hybrid effectiveness-implementation trial [16] designed to test the effectiveness of a standardized OR-to-ICU handoff process while collecting data about the implementation of this process [16]. The project is structured as a parallel mixed methods study with simultaneous collection of qualitative and quantitative data (QUAL + QUAN) during the needs assessment phase (Aim 1). We will use the findings from the needs assessment phase to adapt published handoff processes and test the proposed standard handoff process (Aim 2). We will then implement the standardized handoff, simultaneously collecting both qualitative and quantitative data (equally weighted) to evaluate the effectiveness of the intervention and gather information about the implementation strategy and of the intervention itself (Aim 3). The study draws on the Consolidated Framework for Implementation Research (CFIR), [21] which describes 5 domains to guide implementation research: (1) intervention characteristics, (2) outer setting (economic, political and social context), (3) inner setting (structural, political, cultural context of organizations), (4) individuals involved, and (5) implementation process. Most of these CFIR constructs do not have validated objective measures and thus will be explored using a grounded theory [22] approach.

The methods that we will use to achieve the study aims are detailed below, with CFIR constructs indicated in *italic typeface*.

### AIM #1: Needs assessment of the current handoff process

The OR-to-ICU handoff process involves the physical transfer of patients from the operating room to the intensive care unit as well as the transfer of patient care responsibilities. In both study hospitals, the patient is transported by an anesthetist (physician or certified registered nurse anesthetist) and a member of the surgical team (physician or physician assistant). Patients have real-time physiologic monitoring during this transfer, with heart rate and blood pressure data continuously available to the transporting clinicians who may administer medications or perform other interventions (e.g. cardiopulmonary resuscitation) as needed for life support. On arrival to the intensive care unit, physiologic monitors are transferred to the in-room monitors, patient stability is ensured, and a conversation ensues between the transmitting providers (anesthetist, surgeon) and the receiving providers (physician, nurse practitioner, registered nurse). Although handoff communication commonly takes place at the patient’s bedside, there may be additional asynchronous communication about the patient between the ICU team and members of the surgical team who are not physically present for the handoff. There are no explicit guidelines or expectations about the way that handoff communication should be conducted, but all providers involved in the patient’s physical transfer are expected to engage in the handoff in some way.

#### AIM 1 procedures

Over the course of 8–12 weeks, we will evaluate OR-to-ICU handoffs as they currently take place in two surgical intensive care units (*inner setting)* in Philadelphia, Pennsylvania (*outer setting).* Although these ICUs are part of the same health system, they serve different surgical populations and are staffed by different types of providers (*individuals involved*; Table 1). Patients receiving care in the two study units live throughout the Delaware Valley and represent a diverse payor mix (Medicaid, Medicare, private payors) as well as indigent patients receiving uncompensated care (*outer setting*).

**Table 1:** **Study ICU characteristics**

	Study unit	
Characteristic	Study unit 1	Study unit 2
Surgical specialties represented	General, oncologic, orthopedic, otorhinolaryngologic, plastic, transplant, trauma, urologic, vascular	Cardiac, general, orthopedic, vascular
Beds	24	16
ICU model	Semi-closed*	
Clinicians	• Registered nurses	• Registered nurses
	• Attending physicians: ICU, surgery	• Attending physicians: ICU, surgery
	• Fellows: ICU, surgery	• Fellows: cardiac surgery
	• Residents: anesthesia, ICU, surgery	• Residents: anesthesia, ICU, surgery
	• Advanced practitioners: nurse practitioner	• Advanced practitioners: physician assistants, certified registered nurse anesthetists
Patient demographics	50% white, 49% black, 1% Asian, 9% other	

*“Semi-closed” indicates that each patient in the study units has two care provider teams – one surgical team and one intensive care unit team. Other models include “open” units where patients have just a surgical care provider team and “closed” units where patients have just an ICU provider team.

The needs assessment has four components:

1. Observation of OR-to-ICU handoffs: On notification of patient arrival to the ICU, trained research staff will directly observe the OR-to-ICU handoff by audio recording the handoff and taking detailed field notes about their observations. Research staff will complete a checklist (Additional file 1) capturing elements of handoff content, team performance [10] and handoff quality [23]. Immediately after each handoff, audio recordings will be reviewed to ensure accuracy of checklist completion^a^. Then, the observers will answer open-ended prompts (Additional file 2) about the handoff to guide recording of their impressions. A subset of the handoffs will be observed by two staff members, allowing for calculation of inter-rater reliability of checklist completion. In Study Unit 1, handoffs are sometimes video recorded as part of an ongoing performance improvement project. If these recordings exist, research staff will review them to facilitate interrater calibration and to determine whether they provide additional information about the handoff process. To capture elements of handoff communication not occurring at/near the patient bedside, we will query ICU providers about other sources of patient information, such as telephone conversations and the electronic medical record. This query will be delivered as a self-administered questionnaire completed after the in-person handoff.

(2) Focused chart reviews: We will abstract the pre-, intra-, and postoperative records of each patient whose handoff is observed to enable the research team to determine the accuracy of handoff information transfer with respect to five specific content areas: past medical history, allergies, airway management, medication infusions, and intraoperative fluid balance. We chose these content areas because, to our knowledge, they are present on all published OR-to- ICU handoff checklists.
(3) Event report reviews: With permission from hospital administration, the voluntary event reports of each ICU will be reviewed on a weekly basis to determine whether any reported adverse events were attributed to handoffs. Basic characteristics of any such events will be recorded as research data.
(4) Focus groups, interviews, surveys: We will take several approaches to elicit the thoughts and impressions of clinicians involved in the handoff process (*individuals involved*; Table 2). *Focus groups*: Ten to twelve focus groups will be held in total; these will be evenly split between the needs assessment and evaluation phases. In study unit 1 (Table 1), we will hold separate focus groups of nurses and residents. In study unit 2, we will hold separate focus groups of nurses, residents, and certified registered nurse anesthetists. These focus groups will be led by a trained moderator from the Mixed Methods Research Laboratory at the University of Pennsylvania. The purpose of the focus groups is to elicit perceptions about the current handoff process and impressions about standardized handoffs while another member of the research team takes field notes. The focus groups will be audio recorded and professionally transcribed. *Interviews*: Nurse practitioners and physician assistants in the study ICUs will be individually interviewed because there are relatively few of them and assembling them into one focus group would be logistically challenging. The interviews will be conducted by a single investigator (MBL-F), who will elicit perceptions about the current handoff process and impressions about standardized handoffs. *Surveys*: There are more than 700 clinicians (Table 2) who participate in the OR-to-ICU handoff process. Given that we will be unable to observe most of these clinicians, we designed a survey to ask providers about their current experience with and attitudes about OR-to-ICU handoffs (Additional file 3). All clinicians who participate in OR-to-ICU handoffs will be invited to take this electronic, self-administered questionnaire. Survey data will be collected and managed using REDCap electronic data capture tools [24] hosted at the University of Pennsylvania.

**Table 2:** **Eligible subjects in study population, stratified by study phase**

Study component	Recruitable population*	Target sample size
**Observation (first round)**	440 patients	40-60 patients
	366 clinicians	40-60 handoffs: each consisting of 4-6 clinicians
**Surveys (first round)**	460 clinicians:	460 clinicians
	100 OR nurses	
	100 attending physicians and fellows	
	130 ICU nurses	
	120 residents and CRNAs	
	12 NPs/PAs	
**Interviews (first round)**	12 NPs/PAs	12 NPs/PAs
**Focus groups (first round)**	130 ICU nurses	3 focus groups of 5-8 participants
		(total 15 to 24 ICU nurses)
	120 resident physicians and CRNAs	3 focus groups of 5-8 participants
		(total 15 to 24 residents/CRNAs)
**Simulations**	12 NPs/PAs	4 NPs/PAs (1 per simulation)
	120 residents	8 residents (2 per simulation)
	130 ICU nurses	8 nurses (2 per simulation)
**Observation (second round)**	440 patients	40-60 patients
	366 clinicians	40-60 handoffs consisting of 4-6 clinicians each
**Surveys (second round)**	460 clinicians:	460 clinicians
	100 OR nurses	
	100 attending physicians and fellows	
	130 ICU nurses	
	120 residents and CRNAs	
	12 NPs/PAs	
**Interviews (second round)**	12 NPs/PAs	12 NPs/PAs
**Focus groups (second round)**	130 ICU nurses	2 focus groups of 5-8 participants
		(total 10 to 16 ICU nurses)
	120 resident physicians and CRNAs	2 focus groups of 5-8 participants
		(total 10 to 16 residents/CRNAs)

*Abbreviations: CRNA* certified registered nurse anesthetist, *ICU* intensive care unit, *NP* nurse practitioner, *OR* operating room, *PA* physician assistant.

*Numbers given are estimates, as patient volume and clinician staff numbers are expected to vary over the course of the study.

#### AIM 1 measures

Aim 1 will address the question “How do OR-to-ICU handoffs occur in settings without a standardized process?” As we collect data to answer this question, we will also collect information to enable development of a standard handoff process. Given the hybrid nature of the study, we will measure concepts related to both effectiveness and implementation (Table 3). These are not true “outcomes” because they are collected at baseline, but they will enable us to detect changes occurring in the intervention phase:

**Table 3:** **Study outcomes stratified by measure type (implementation vs. intervention) and data type (qualitative vs. quantitative)**

		Measure type	
		*Implementation*	*Intervention*
Data type	*Qualitative*	Acceptability (primary)	Handoff quality
		Appropriateness	
		Fidelity	
		Sustainability	
	*Quantitative*	Acceptability*	Information omissions, number (primary)
			Handoff accuracy
			Handoff duration
			Team members present, number
			Teamwork quality**
		Fidelity	Professionalism
			Diagnostic test utilization
			Medication orders
			Patient ICU length of stay
			Patient ICU mortality
			Patient hospital mortality

*Assessed with 5-point Likert scale.

**Assessed with 3-point scale.

Effectiveness outcome 1: Number of information omissions. Published studies demonstrate that important patient information is frequently omitted from OR-to-ICU handoff communication when these handoffs are not standardized [8, 12]. Using a data capture tool developed for this project (Additional file 1), we will determine how many data elements are absent from each handoff.

Effectiveness outcome 2: Handoff accuracy. To our knowledge, no studies to date have reported whether the information reported during OR-to-ICU handoff is actually true. As such, there are no validated measures of handoff accuracy. To attempt to determine whether OR-to-ICU handoffs are accurate, we will compare observed handoff content (for the five elements described above) to the patient’s medical record to calculate accuracy. Accuracy will be defined as the number of content areas correctly relayed during handoff divided by the number of content areas for which medical record data is available.

Additional effectiveness outcomes: We will collect data about patient outcomes, including ICU and hospital mortality as well as ICU and hospital length of stay. We will collect data about resource utilization in the first 24 hours of ICU admission (e.g. diagnostic test utilization, medications). We will also measure process outcomes such as number of team members present, handoff duration, quality of teamwork (coded on a 3-point scale), and professionalism (coded on a 3-point scale).

Implementation outcome 1: Acceptability, defined as “the perception among implementation stakeholders that a given treatment, service, practice, or innovation is agreeable, palatable, or satisfactory” [25]. We know of no validated measures of acceptability in critical care or perioperative settings. Investigators in mental health have used semi-structured interviews and questionnaires to assess acceptability [25]. For our study, we will analyze survey data and interview/focus group transcripts to qualitatively characterize acceptability of the current handoff practice and that of a proposed standardized process. Drawing on CFIR, we will explore how the inner setting of each ICU and the individuals involved impact acceptability of a standardized handoff process as well as the other implementation constructs described below.

Additional implementation outcomes: Appropriateness, defined as the “perceived fit, relevance, or compatibility” of a standardized handoff process, and feasibility, defined as the extent to which a standardized handoff process can be carried out [25]. As with “acceptability”, we will qualitatively characterize these implementation outcomes.

### AIM #2: Implementation of a standardized OR-to-ICU handoff process

**Figure 2:**
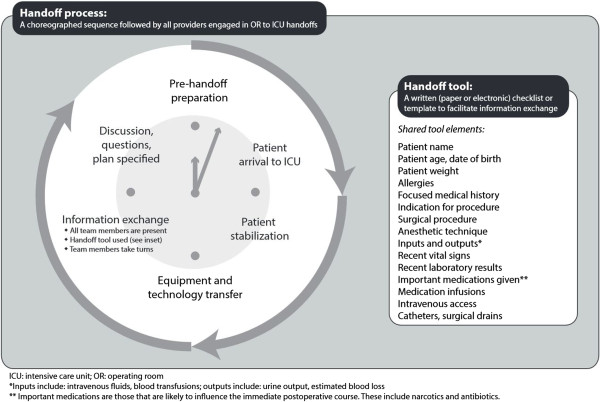
**Shared features of OR-to-ICU handoffs in published studies.**

The published literature contains both tools (i.e. checklists, forms) [5, 7–9, 11, 12] and protocols (i.e. pathways, care algorithms) [7–9] [10] [26] used in standardized OR-to-ICU handoffs. There are common elements of both the tools and the protocols (Figure 2).

#### AIM 2 procedures

On the basis of the needs assessment (Aim #1), and in conjunction with ICU clinical leadership (i.e. nurse managers, medical directors), the research team will adapt published protocols to develop a standard process incorporating the informational needs and clinical priorities of the clinicians who will employ it. This implementation process will include the development of a customized tool or checklist to facilitate information transfer. We will take care to account for the clinical priorities of disparate surgical populations, developing a tool that balances the amount of information provided (enough to facilitate care and anticipate patient needs) with the length of the instrument (not so long as to be burdensome).

Once we have developed a standardized process, we will conduct in situ (i.e. in the hospital) simulations of this process to determine whether the process, as planned, is a reasonable replacement for the current OR-to-ICU workflow. Trained staff from the Penn Medicine Simulation Center will facilitate simulation sessions to elicit feedback from participants for the candidate standardized process. Realistic manikins will be programmed to exhibit vital signs and will be connected to transport monitors so that we can closely mimic the transport process of a critically ill patient. The manikins will be transported to the ICU by clinicians from anesthesia and surgery. This mock OR team will then carry out the new handoff process with a mock ICU team consisting of an ICU registered nurse and a nurse practitioner or resident physician. During the simulated handoff, trained observers will score the interaction using the tool employed in Aim 1. After simulating the standardized process, a debriefing session will be held to elicit feedback about the process. Simulations will be video recorded and debriefing sessions will be audio recorded; we will analyze these recordings to find themes relating to intervention feasibility and provider acceptance of the proposed handoff intervention.

The full-scale implementation process will employ 4 of 6 published implementation strategy categories described by Powell *et al.* [27] (*implementation process*)^b^. The first 3 strategies are part of Aim 2, and are described here.

Implementation Strategy 1 - Planning: The needs assessment (Aim 1) described earlier is the first part of the planning process. Once we analyze data from the needs assessment, we will share our findings with physician and nurse leaders in each ICU (“champions”). We will work with these champions to create a new handoff process that incorporates elements from published studies (Figure 2) as well as important themes emerging from the needs assessment (Aim 2). We will also work with ICU champions to troubleshoot problems with implementation. Anticipated problems with implementation include the following: tool availability and ease of use, patient physiologic instability distracting from the process, clinician familiarity with the tool and the process, and clinician reluctance to adopt a new process. The research team will take detailed field notes during planning sessions with local champions.

Implementation Strategy 2 - Education: We will hold meetings of nursing and physician staff to share the results of the needs assessment and process development and to share the plan for implementation. Clinicians’ concerns will be addressed during these meetings and recorded with field notes taken by the research team. The handoff process will be adapted to account for these concerns. The research team will develop a glossary of terms related to the handoff process that will serve as an enduring reference, and the research team will be available in- person, by telephone, and via electronic mail during the study period for consultation as needed. We have created a study website (http://www.pennhatricc.com) that will serve as an additional educational resource for clinical staff and anyone with questions related to the study.

Implementation Strategy 3 - Restructuring: We will facilitate relay of clinical data from the OR team to the ICU team by making the handoff tool part of the patient’s bedside (nursing) chart. In this way, written information will be available to supplement and reinforce the verbal information transmitted during the handoff process. We will work with our local champions to facilitate this restructuring.

#### AIM 2 measures

Given that Aim 2 precedes use of a standard handoff process, we will assess only implementation outcomes for this phase of the study.

Implementation outcome 1: Acceptability of a standard handoff process. Once findings from the needs assessment are shared with providers and clinical leadership, perceptions of our proposed intervention may change. We will assess acceptability as described above, using simulation debriefing transcripts and field notes from interactions with clinical champions.

Additional implementation outcomes: Appropriateness and feasibility, assessed qualitatively as described above.

### AIM #3: Evaluate the implementation and effectiveness of a standardized OR- to-ICU handoff process

#### AIM 3 procedures

As mentioned above, the full-scale implementation process employs 4 of 6 implementation strategy categories described by Powell *et al.* [27] (*implementation process*). Aim 3 incorporates the fourth implementation strategy.

Implementation Strategy 4 – Quality Management: After implementation, the research team will evaluate the new handoff process using the methodology employed in the needs assessment phase. We will repeat the data collection procedures described for Aim 1 - observations, chart reviews, event report reviews, focus groups, interviews and surveys. We will use the same instruments for observations and chart reviews for Aims 1 and 3. In the focus groups, interviews and surveys for this Aim, questions and probes will elicit impressions about the new handoff process, including feasibility and acceptance of the new process.

The results of these assessments will be shared with clinical staff via meetings and poster boards in each ICU. After the initial evaluation period, we will evaluate 10 handoffs monthly for one year to test the durability of the intervention. Based on findings from these late evaluations, we will re-adapt the tool and process as needed.

#### AIM 3 measures

In the “pre-post” design of this study, Aim 1 measures represent the pre-intervention period and Aim 3 measures represent the post-intervention period. The Aim 3 procedures, therefore, mirror those for Aim 1. The measures are also the same: number of information omissions, handoff accuracy, patient ICU/hospital length of stay and mortality, resource utilization, number of team members present for handoff, handoff duration, quality of teamwork and professionalism (effectiveness); acceptability, appropriateness and feasibility (implementation).

We will also collect data about fidelity, another implementation outcome without a relevant validated measure. Fidelity is generally described as the “faithful” use of a treatment or intervention, or the adherence to the “essential” components of an intervention [28]. For this study, we will create a rating scheme to enable different raters to characterize adherence to elements of the standardized handoff (e.g. team members present, use of a structured form or tool). We will characterize fidelity as the percentage of handoff elements present in a given handoff observation.

### Sample size and power considerations

**Figure 3:**
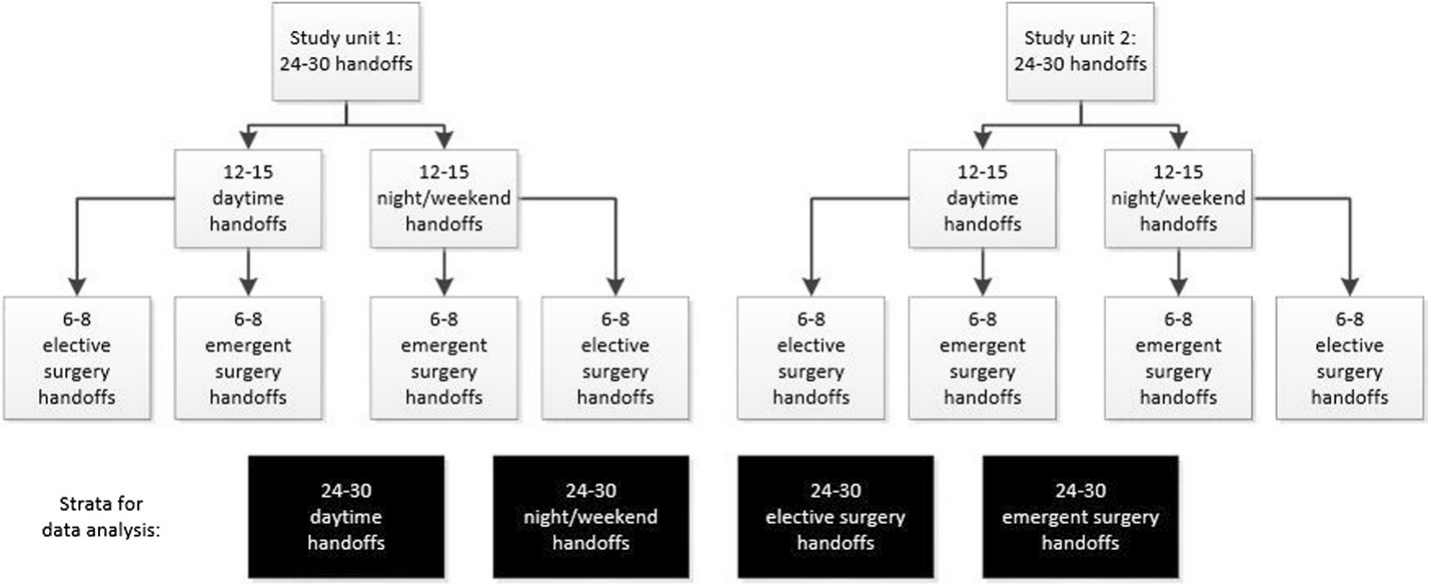
**Handoff observation stratification scheme.**

Qualitative data: Thematic saturation is the point at which additional observations add little or no additional information about the phenomenon of interest [29]. In ethnographic studies, thematic saturation is typically observed after 12–15 observations [30]. Our prior experience suggests that handoffs occurring during the day may materially differ from those conducted at night, so we will collect at least 12 handoffs during the day and 12 during nights and weekends. Additionally, handoffs for patients undergoing elective surgery may be different from patients undergoing emergency surgery. We will also stratify observations by this criterion (Figure 3). Consequently, we expect to reach thematic saturation after observing 48-60 handoffs. The handoffs will be evenly distributed across the two study sites.

Quantitative data: As the study is a Type 1 hybrid effectiveness-implementation trial, [16] power calculations are based on our effectiveness hypothesis. Previously published work suggests that, on average, 36-40% of handoff information is omitted in the absence of a standardized handoff process [7, 8]. After institution of a standardized process, information omissions drop by 50-66% [7, 8]. Our handoff observation tool has 13 content items; if our observations are consistent with published work, we will observe 4–5 omissions per handoff episode. We are assuming a standard deviation of 2 information omissions given the heterogeneity of this process, although others have reported a smaller standard deviation [7]. If these assumptions hold true, we will have 95.7% power to detect a drop in information omissions from 4.5 to 3 (a 33% drop), based on a sample size of 96 (48 pre-intervention, 48 post-intervention), and a two-tailed alpha of 0.05. The power calculation suggests that our target sample size for qualitative data will also be sufficient to detect pre- and post- intervention differences in our primary quantitative outcome (Table 3).

### Data analysis

Qualitative approach: We will use a grounded theory approach [22] to develop a codebook and to analyze qualitative data (transcripts from interviews and focus groups, field notes, video recordings). During the analytic process, we will consider how the CFIR constructs described above affect our effectiveness and implementation outcomes. We will also probe for differences between the two study sites that may impact the intervention’s implementation or effectiveness. NVivo 9.0 (QSR International, Doncaster, Victoria, Australia) will be used to manage qualitative data and to facilitate analysis. Qualitative data will be coded by two research personnel; disagreements will be adjudicated by a third person on the research team. We will use the inter-rater reliability function in NVivo to assure consistency in coding within and across coders. Data analysis will start during the data collection for Study Aim 1, allowing us to adjust data collection procedures to adequately explore emerging themes.

Quantitative approach: We will use descriptive statistics to characterize quantitative measures (Table 3). We will stratify our analysis by study unit, time of handoff (day versus night/weekend), elective versus emergency surgery, and surgical specialty. To test the hypothesis about changes in information omissions, we will conduct a Mann–Whitney U test comparing means before and after the intervention. In addition to comparing information omissions within ICUs before and after standardization of the handoff process, we will compare patient outcomes (ICU length of stay, ICU mortality, hospital mortality) in these two study units with contemporary outcomes from two similar non-study units within the same healthcare system. Using a difference-in-differences approach, this latter comparison will enable us to gauge the likelihood that changes in patient outcomes over time were attributable to our intervention or to secular trends. For the quantitative implementation outcomes acceptability and fidelity, we will the Mann Whitney U test to detect site differences in intervention acceptability and fidelity. Quantitative data analyses will be conducted using the Stata program (version 12, StataCorp LLC, College Station, Texas).

Mixed methods approach: We will convene at least three members of the research team to characterize observed handoffs in Aims 1 and 3 as “satisfactory” or “unsatisfactory” on the basis of field notes and data from our handoff checklists. Once handoffs have been categorized in this way, we will re-analyze our quantitative outcomes (mortality, length of stay, resource utilization, etc.), stratifying them by the quality of handoff. We will perform this analysis with Aim 1 data alone to study the relationship between “good” handoffs and patient outcomes in the absence of a standard handoff process, and will also perform this analysis again once we have Aim 3 data.

#### Study status

HATRICC has been reviewed and approved by the Institutional Review Board of the University of Pennsylvania (study number 819726). Information-sharing sessions have been held with the physician and nurse leaders of the study units, and we have presented the study at educational conferences for surgery and anesthesia. Data collection commenced on July 2, 2014. Data analysis is projected to start at the end of August 2014.

## Discussion

The HATRICC study will examine the effectiveness and implementation of a standardized OR-to-ICU handoff process in two intensive care units currently without a standardized process. This study represents an important step in understanding how to apply the findings of studies suggesting that standardized handoff processes improve information exchange and patient outcomes.

To date, studies of OR-to-ICU handoffs have focused on the effectiveness of interventions to standardize this process. However, implementing such a complex intervention (Figure 2) requires a systematic approach, including identification of local champions, [5, 27] development of an acceptable protocol, [4] and clinician engagement and education [4, 5, 7–10, 27]. Most published reports in this field limit description of the implementation process to a few statements in the methods sections, leaving unexplained crucial details about how to make the process actually work. The HATRICC study will provide a template for implementing complex clinical interventions and evaluating the effectiveness of those interventions. In the event that our intervention is ineffective in improving information exchange and/or patient outcomes, the detailed needs assessment and implementation findings will shed light on potential alternative approaches to improving handoffs.

This project has important limitations: First, the study is designed as a pre-post intervention. As such, there is no true control that will allow us to determine whether changes in communication and patient outcomes can be attributed to our intervention. To improve our ability to identify whether our intervention is effective, we will stagger the timing of implementation between the two study units. We will also collect patient data about length of stay and mortality from similar non-study units within our health system, which will allow us to determine whether important secular changes have occurred that might explain our findings. Second, we do not have validated instruments for our implementation metrics. Concepts such as acceptability and fidelity are commonly assessed in implementation studies but validated measures with demonstrated criterion validity are lacking [31]. Creating validated instruments is outside the scope of the current project, so we plan to assess implementation outcomes with a largely qualitative approach. There is a precedent for using qualitative data to characterize implementation outcomes, [25] but we understand that numerical representations of these concepts are more readily understood. Therefore, we will collect quantitative data on fidelity and “semi-quantitative” data on acceptability, asking providers to assess this with a Likert scale (Table 4). Finally, although we will be collecting detailed information about the implementation process, this is not a trial of implementation strategies. We will not, therefore, have information about the minimum necessary implementation approaches needed to achieve similar outcomes. The findings from this study will constitute pilot data for our future studies of implementation strategies.

**Table 4:** Implementation strategy categories and specific approaches*

Planning	Education	Restructuring	Quality mgmt.
• Needs assessment (SA1)	• Implementation glossary	• Facilitate relay of clinical data to providers	• Audit and provide feedback
• Prepare champions	• Educational meetings		• Purposely re-examine implementation
• Formal blueprint			
• Consensus discussions	• Ongoing consultation		

*Categories from Powell *et al*. [27].

There are several strengths to this project as well. First, the hybrid effectiveness- implementation structure will enable us to contribute to the body of scientific work about improving postoperative communication while offering implementation findings that may be of use to other investigators. Second, the mixed methods approach will allow us to develop a comprehensive understanding of the handoff process from the perspectives of all the clinicians involved, improving our ability to develop and implement a feasible and acceptable intervention. Third, collecting data on both process outcomes (e.g. information omissions, teamwork quality) and patient outcomes (e.g. mortality, length of stay), will allow us to test the relationship between handoff quality and patient outcomes, a relationship that is assumed but not explicitly tested in many handoff studies. Lastly, the focus on a mixed surgical population extends the scope of work done by other investigators, increasing the potential impact of this work.

## Endnotes

^a^Audio recordings will not be transcribed and formally coded because noise artifacts are commonly present. In lieu of formal coding of audio data, research staff members will reflect on the contents of the audio recording during their post-observation reflections.

^b^We omitted the financing and policy strategies described by Powell because they did not seem to be relevant to implementation on a small scale (i.e. two hospital wards). In future larger studies, we plan to incorporate these strategies as well.

## Electronic supplementary material

Additional file 1: **Handoff observation tool (checklist).** (PDF 1 MB)

Additional file 2: **Handoff observation open-ended questions.** (PDF 546 KB)

Additional file 3: **Pre-intervention survey.** (PDF 487 KB)

## Abbreviations

HATRICC: Handoffs and Transitions in Critical Care
ICU: Intensive Care Unit
NP: Nurse Practitioner
OR: Operating room
PA: Physician assistant
RN: Registered nurse.
