# Supplementary material for: Handoffs and transitions in critical care (HATRICC): protocol for a mixed methods study of operating room to intensive care unit handoffs
Source: BMC Surg. 2014 Nov 19;14:96. doi: 10.1186/1471-2482-14-96 (PMC4255652; doi:10.1186/1471-2482-14-96)
Supplement: Supplementary file 1 — Additional file 1: Handoff observation tool (checklist). (PDF 1 MB) [file 12893_2014_535_MOESM1_ESM.pdf]

ICU Handoff Assessment Tool

Date:
Time start:
Observer:
Room:

PATIENT STICKER HERE
NAME:
MRN:
DOB: **SEX: F M**

Stability: Stable Unstable RRT/Code
Admission type: Planned/elective Unplanned/Urgent
New ICU admission? New ICU re-admit
Origin: OR ED ward other ICU OSH Other:___________
Receiving: HUP Rh5 PPMC M2 Other: _________
Number of people in room: __________

Handoff types:
- ☐ Anes MD/CRNA to ICU MD/NP
- ☐ Anes MD/CRNA to ICU RN
- ☐ Anes MD/CRNA to resp tech
- ☐ Surg MD/PA to ICU MD/NP
- ☐ Surg MD/PA to ICU RN
- ☐ Origin RN to ICU RN
- ☐__________ to whole room
- ☐__________ to ___________
- ☐__________ to ___________

HANDOFF CONTENT

Hands-on exam by receiving team?	Y☐	N☐	Unk☐	Exam
Past medical history	Y☐	N☐	Unk☐	PHist
Reason for ICU admission	Y☐	N☐	Unk☐	HPI
Allergies	Y☐	N☐	Unk☐	Allergies
Airway	Y☐	N☐	Unk☐	Airway
Breathing/ventilation	Y☐	N☐	Unk☐	Breathing
Circulation/hemodynamics	Y☐	N☐	Unk☐	Circ
Inputs	Y☐	N☐	Unk☐	Ins
Outputs	Y☐	N☐	Unk☐	Outs
Drains/lines	Y☐	N☐	Unk☐	Lines
Complications (or absence of)	Y☐	N☐	Unk☐	Comps
Plan	Y☐	N☐	Unk☐	Plan
Family	Y☐	N☐	Unk☐	Family
Team contact info	Y☐	N☐	Unk☐	Contact

PMH/SurgHx:
- ☐HTN
- ☐CAD / MI
- ☐s/p CABG
- ☐DM
- ☐Renal failure

Allergies:
Y☐ N☐
List allergies:

Airway:
- ☐ "Difficult"
- ☐ Fiberoptic
- ☐ "Easy"

Vasoactives:
- ☐ Phenylephrine ("Neo")
- ☐ Norepi ("Levophed")
- ☐ Epinephrine ("Epi")
- ☐ Vasopressin ("Vaso")
- ☐ Nicardipine ("Cardene")
- ☐ Nitroglycerin ("Nitro")
- ☐ Other:

Inputs:______________________

Outputs:______________________

COMMUNICATION QUALITY, SETTING

Trans Provider: Rec Provider:
Handoff Start: ___________ Stop: ___________
Location: bedside hallway phone other:______
If in-person handoff, complete remainder of box

*Quality of **Transmitting** Provider Delivery:*
Superior Satisfactory Unsatisfactory

*Attributes of **Recipient** Listening:*

Eye contact	Y☐	N☐
Affirm. statements	Y☐	N☐
Head nodding	Y☐	N☐
Note-taking	Y☐	N☐
?-asking/interactive	Y☐	N☐

Noise: Quiet Neither quiet nor loud Loud
Interruption Score (see reverse for grading): ____
unrelated to patient care (e.g. pages): ____
due to patient care: ____

Trans Provider: Rec Provider:
Handoff Start: ___________ Stop: ___________
Location: bedside hallway phone other:______
If in-person handoff, complete remainder of box

*Quality of **Transmitting** Provider Delivery:*
Superior Satisfactory Unsatisfactory

*Attributes of **Recipient** Listening:*

Eye contact	Y☐	N☐
Affirm. statements	Y☐	N☐
Head nodding	Y☐	N☐
Note-taking	Y☐	N☐
?-asking/interactive	Y☐	N☐

Noise: Quiet Neither quiet nor loud Loud
Interruption Score (see reverse for grading): ____
unrelated to patient care (e.g. pages): ____
due to patient care: ____

Quality of Teamwork: Superior Satisfactory Unsatisfactory **Quality of Professionalism:** Superior Satisfactory Unsatisfactory

Provider presence: Time all providers gone: ________ Time all nurses gone: ________ Time all clinical staff gone: ________

APACHE 2 Score: _______

Version 3 | Tool last updated: 30 June 2014

*** FOR RESEARCH ONLY ***
DO NOT FILE IN MEDICAL RECORD
