# Supplementary material for: Handoffs and transitions in critical care (HATRICC): protocol for a mixed methods study of operating room to intensive care unit handoffs
Source: BMC Surg. 2014 Nov 19;14:96. doi: 10.1186/1471-2482-14-96 (PMC4255652; doi:10.1186/1471-2482-14-96)
Supplement: Supplementary file 2 — Additional file 2: Handoff observation open-ended questions. (PDF 546 KB) [file 12893_2014_535_MOESM2_ESM.pdf]

HATRICC
Handoffs and Transitions in Critical Care

Handoff observation open-ended questions

(Before discussion)

1) **Describe the interactions between handoff participants.** (Did people seem to know each other? Did the participants use each other's names? Were people paying attention?)

2) **Describe provider engagement.** (Did the participants seem interested, engaged, paying attention? How do you know?)

3) **Describe the actors in the handoff scene.** (Who was present? What was their role? How do you know what each person's role was? Consider body language, attire, interactions with others.)

4) **How was handoff information relayed?** (How was the information organized? How would you describe the communication styles used? How much information was offered by handoff provider vs. prompted by recipient?)

5) **Explain why you scored quality of teamwork the way that you did.**

6) **Explain why you scored quality of professionalism the way that you did.**

7) **Describe the context of the handoff.** (Where is handoff occurring? What else is going on at the same time? Consider what is happening in the room as well as what is going on in the ICU in general.)

8) **What was unusual about this handoff, if anything?**

9) **Did the audio recording help? If not, why not? If yes, why?**

(After discussion)

10) **Describe any additional impressions you developed after discussion with your co-observer.**

General notes:

- If making a subjective assessment (e.g. "The handoff didn't go well."), be sure to explain why you came to this conclusion. Use explicit descriptions of behavior to justify your conclusions.
- Consider body language in each of the responses.
