# Supplementary material for: Handoffs and transitions in critical care (HATRICC): protocol for a mixed methods study of operating room to intensive care unit handoffs
Source: BMC Surg. 2014 Nov 19;14:96. doi: 10.1186/1471-2482-14-96 (PMC4255652; doi:10.1186/1471-2482-14-96)
Supplement: Supplementary file 3 — Additional file 3: Pre-intervention survey. (PDF 487 KB) [file 12893_2014_535_MOESM3_ESM.pdf]

OR to ICU handoff study

Please complete the survey below.

Thank you!

1) What kind of care provider are you?
- ☐ Physician
- ☐ Nurse (CNA, RN)
- ☐ Advanced practitioner (CRNA, NP, PA)
- ☐ Student
- ☐ Other

2) What physician role do you have?
- ☐ Attending physician
- ☐ Fellow
- ☐ Resident or intern

3) What nurse role do you have?
- ☐ ICU nurse (RN)
- ☐ OR nurse (RN)
- ☐ CNA
- ☐ Other type of nurse

4) What kind of advanced practitioner are you?
- ☐ CRNA
- ☐ NP
- ☐ PA

5) You selected "Other" as your provider type. What kind of provider are you?

____

6) In what clinical locations do you work? Choose all that apply.
- ☐ HUP Rhoads 5 SICU
- ☐ HUP OR
- ☐ PPMC Myrin 2 SICU
- ☐ PPMC OR

7) Do you think of yourself as someone who GIVES OR-to-ICU handoffs, someone who RECEIVES them, or both?
- ☐ I am part of the OR team GIVING handoffs to the ICU team.
- ☐ I am part of the ICU team RECEIVING handoffs from the OR team.
- ☐ BOTH: I am sometimes part of the OR team and sometimes part of the ICU team.

8) List some words or short phrases that come to mind when you think about OR to ICU handoffs.

____

9) Move the slider to indicate your current SATISFACTION with OR to ICU handoffs.

Very dissatisfied	Neither satisfied nor dissatisfied	Very satisfied

(Place a mark on the scale above)

How often are the following statements true?

	Never	Rarely	Sometimes	Usually	Always
10) I feel comfortable handing off to other providers.	☐	☐	☐	☐	☐
11) During handoff, I transmit all important patient information.	☐	☐	☐	☐	☐

12)

	I know what information the receiving care provider needs.	☐	☐	☐	☐	☐
13)	During handoff, I ask the receiving provider if they have questions.	☐	☐	☐	☐	☐
14)	During handoff, I sometimes forget to mention something important.	☐	☐	☐	☐	☐
15)	I think that patient handoffs are important.	☐	☐	☐	☐	☐
16)	I use the Epic critical care handoff tool.	☐	☐	☐	☐	☐
17)	I need to access the medical record to get information that should have been transmitted in handoff.	☐	☐	☐	☐	☐
18)	I have to find other clinicians to get information that should have been transmitted in handoff.	☐	☐	☐	☐	☐
19)	I don't know the person I am handing off to/from.	☐	☐	☐	☐	☐
20)	I am happy with the current way handoffs are conducted.	☐	☐	☐	☐	☐

21) Would any of the following make you consider changing how you perform your handoffs? Choose all that apply.

- ☐ Nothing - I am satisfied with my current practice
- ☐ Evidence that a different process improves COMMUNICATION
- ☐ Evidence that a different process improves PATIENT OUTCOMES
- ☐ Data showing that UPHS patients are harmed by handoffs
- ☐ Data showing that UPHS perioperative handoffs show poor communication
- ☐ Other reason

22) You indicated "Other reason" for the question above. What other reasons would make you consider changing how you perform your handoffs?

____

23) Do you want to be entered into a lottery to win a $50 Starbucks or Amazon gift card?

- ☐ Yes
- ☐ No

24) Are you interested in being contacted about an interview or focus group? You will be paid for participation.

- ☐ Yes
- ☐ No

25) Are you interested in being contacted about participating in a simulation of a handoff in the ICU? You will be paid for your participation.

- ☐ Yes
- ☐ No

26) Please provide your email address.

____
